# Interaction of Kinase-Interaction-Motif Protein Tyrosine Phosphatases with the Mitogen-Activated Protein Kinase ERK2

**DOI:** 10.1371/journal.pone.0091934

**Published:** 2014-03-17

**Authors:** Dana M. Francis, Dorothy Koveal, Antoni Tortajada, Rebecca Page, Wolfgang Peti

**Affiliations:** 1 Department of Molecular Pharmacology, Physiology and Biotechnology, Brown University, Providence, Rhode Island, United States of America; 2 Department of Molecular Biology, Cell Biology and Biochemistry, Brown University, Providence, Rhode Island, United States of America; 3 Department of Chemistry, Brown University, Providence, Rhode Island, United States of America; George Washington University, United States of America

## Abstract

The mitogen-activation protein kinase ERK2 is tightly regulated by multiple phosphatases, including those of the kinase interaction motif (KIM) PTP family (STEP, PTPSL and HePTP). Here, we use small angle X-ray scattering (SAXS) and isothermal titration calorimetry (ITC) to show that the ERK2:STEP complex is compact and that residues outside the canonical KIM motif of STEP contribute to ERK2 binding. Furthermore, we analyzed the interaction of PTPSL with ERK2 showing that residues outside of the canonical KIM motif also contribute to ERK2 binding. The integration of this work with previous studies provides a quantitative and structural map of how the members of a single family of regulators, the KIM-PTPs, differentially interact with their corresponding MAPKs, ERK2 and p38α.

## Introduction

The mitogen-activation protein kinases (MAPKs; ERK, p38 and JNK) are cytosolic serine/threonine-specific kinases that coordinate the cellular response to a range of extracellular stimuli. Their activity is tightly regulated by the coordinated action of activating kinases (the MAP kinase kinases), deactivating phosphatases (i.e., the kinase interaction motif protein tyrosine phosphatases or KIM-PTPs, the dual specificity MAPK phosphatases or MKPs, among others) and scaffolding/targeting proteins, which determine their subcellular location [1]. Once activated, the MAPKs phosphorylate a variety of substrates, which ultimately determine the cellular response [2]. Consequently, disruptions in MAPK signaling are correlated with multiple diseases, including cancer, autoimmunity and Alzheimer's disease, among others [3].

MAPKs have a bi-lobed architecture, with a five-stranded β-sheet in the N-terminal lobe and six α-helices in the C-terminal lobe [4], [5]. During the last two decades, cellular and structural studies have revealed that MAPKs contain a conserved docking groove, characterized by two distinct subsites (one charged, Ψ_chg_, and the second hydrophobic, Φ_hyd_) [6]–[8]. Most regulators and substrates bind their cognate MAPKs at this groove via linear peptides, known as KIM- or D-site motifs (Kinase Interaction Motif), with the consensus sequence (R/K)_2-3_-X_2-6_-Φ_A_-X-Φ_B_ (Φ is any hydrophobic residue) [9]–[11]. One family of regulators that bind MAPKs via a KIM is the KIM-PTPs (Kinase Interaction Motif – Protein Tyrosine Phosphatases) [11]–[13]. Members of this family include STEP (PTPN5, striatal enriched protein tyrosine phosphatase), PTPSL/PTPBR7 (PTPRR, PTP STEP-like) and HePTP (PTPN7, hematopoietic protein tyrosine phosphatase). They regulate ERK2 (extracellular signal-regulated kinase 2) and p38α by selectively dephosphorylating their activation loop tyrosines (active state complexes) and also by sequestering the kinases in the cytosol (resting state complexes).

Over the last years different groups, including ours, have studied the interaction of MAPK regulatory proteins, as well as substrates, with different MAPKs, especially ERK2, p38 and JNK [12]–[17]. However, while a plethora of cell biological data is available, much less systematic interaction data using quantitative methods, such as isothermal titration calorimetry (ITC), has been reported. This is of special interest, as recent studies have shown that while KIM-peptides derived from KIM-containing regulatory proteins and substrates readily discriminate between ERK2/p38 and JNK, they discriminate poorly between ERK2 and p38 [7]. We recently reported detailed ITC, NMR as well as small angle X-ray scattering (SAXS) data of the interactions between the MAPK p38α and the KIM-PTP family members [14], [15]. Furthermore, we also compared the interaction the immune-specific KIM-PTP, HePTP, with p38α and ERK2 [16], [17]. These studies showed that HePTP (residues 15–339) as well as the HePTP KIM-peptide (residues 15–31) consistently bind more tightly to ERK2 (∼7-times and ∼4-times, respectively) than p38α. To better understand the molecular determinants that allow regulatory proteins to discriminate between ERK2 and p38α, we investigated the interaction of PTPSL and STEP with ERK2 and then compared them with our previous study examining the same interactions with p38α. Our data shows that both PTPSL and STEP also bind more strongly to ERK2. Furthermore, we find that within the family both STEP and PTPSL interact with ERK2 and p38α more strongly than does HePTP. To determine if this discrimination between p38 and ERK2 is specific for this regulatory protein family, we also compared the interaction of a MAP Kinase Phosphatase (MKP; DUSP16) MAPK binding domain with p38α [18] and ERK2. In contrast to what we observed for the KIM-PTPs, DUSP16 binds both MAPKs with similar affinities, demonstrating that other families of MAPK regulator proteins likely have distinct discrimination factors between the MAPKs. Finally, we correlated these differences with the low-resolution structures of the complexes using small angle x-ray scattering (SAXS) experiments. Collectively, our data is providing a coherent understanding of how subtle differences between the KIM-PTPs contribute to MAPK selectivity and, in turn, MAPK signal fidelity.

## Experimental Procedures

### Peptide and protein preparation

The PTPSL_KIM_ peptide (residues 331–348), STEP_KIM_ (residues 213–229) and STEP_KIMKIS_ (residues 213–256) were synthesized, HPLC purified and verified by mass spectrometry (MS) (>98% purity; Biosynthesis, Inc.). Expression and purification of ERK2 and PTPSL_KIMKIS_ (residues 332–373) was carried out as previously described [14], [15]. PTPSL (residues 332–655, human), STEP (residues 213–539, mouse) and PS_Chimera_ (PTPSL residues 332–373 with STEP residues 257–539; gene synthesized by DNA 2.0) were expressed and purified using the protocol previously described in detail for STEP_CAT_ (residues 244–539) [19]. The DUSP16 MAPK binding domain (residues 5–138, human) was expressed and purified as previously described [18]. All purified proteins were stored at −80 °C until use at which point they were thawed and exchanged into the appropriate buffer using size exclusion chromatography (SEC; Superdex 75 26/60; GE Healthcare). The final protein buffer was dependent on the experiment performed. For ITC experiments, the final SEC purification was performed in ITC buffer (10 mM Tris pH 7.5, 150 mM NaCl, 0.1 mM EDTA, 0.5 mM TCEP); for SAXS experiments, the final SEC purification was performed in SAXS buffer (50 mM HEPES pH 6.8, 150 mM NaCl, 5 mM dithiothreitol). PTPSL_KIM_, STEP_KIM_ and STEP_KIMKIS_ peptides were solubilized in ITC buffer prior to ITC measurements.

### Isothermal Titration Calorimetry

ITC experiments were performed at 25 °C using a VP-ITC microcalorimeter (Microcal Inc.). Titrant (10 μL per injection) was injected into the sample cell over a period of 20 seconds with a 250 second interval between titrations to allow for complete equilibration and baseline recovery. 28 injections were delivered during each experiment and the solution in the sample cell was stirred at 307 rpm to ensure rapid mixing. To determine the thermodynamic parameters (Δ*H*, Δ*S*, Δ*G*) and binding constants (*K*) of the ERK2:PTPSL_KIM_, ERK2:PTPSL, ERK2:STEP and ERK2:PSChimera interactions, PTPSL_KIM_, PTPSL, STEP and PSChimera were titrated into ERK2. To determine the same parameters for the ERK2:STEP_KIM_, ERK2:PTPSL_KIMKIS_ and ERK2:STEP_KIMKIS_ interaction, ERK2 was titrated into the peptides. Titrants were chosen based on the amount of purified protein/peptide available for the experiment. For all titrations the concentrations were slightly varied to ensure independence of the K_d_ from the protein/peptide concentrations. Data were analyzed with a one-site binding model assuming a binding stoichiometry of 1∶1 using Origin 7.0 software. A nonlinear least-squares algorithm and the titrant and sample cell concentrations were used to fit the heat flow per injection to an equilibrium binding equation, providing values of the stoichiometry (*n*), change in enthalpy (Δ*H*) and binding constant (*K*). All data were repeated in triplicate.

### Small angle X-ray scattering

To form the ERK2:STEP resting-state complex, purified, unphosphorylated ERK2 was incubated in a 1∶1 molar ratio with purified STEP_213-539_ at room temperature for 30 minutes and purified using SEC (Superdex 75 26/60 [GE Healthcare]; SAXS buffer). Data for the ERK2:STEP complex were recorded on samples at 0.7, 1.2 and 1.7 mg/ml. All samples were prepared within 48 hours of data acquisition and stored on ice at 4 °C. All samples were filtered (0.02 μM filter, Whatman) immediately prior to data collection. All data was recorded at Beamline X9A at the National Synchrotron Light Source (NSLS) using a Dectris Pilatus 300k (3.4 m distance from the sample for SAXS) and a Photonic Science CCD (0.47 m from the sample for WAXS) detector. 20 μl of sample was continuously flowed through a 1 mm diameter capillary and exposed to an X-ray beam for 30 seconds. Normalization for beam intensity, buffer subtraction and merging of the data from both detectors were carried out using PRIMUS [20]. A Guinier approximation, I(q) = I(0)exp(-q^2^R_g_^2^/3), where a plot of ln[I(q)] and q^2^ is linear for q<1.3/R_g_ (q is the scattering vector q = 4πsin(θ)/λ; λ, wavelength of X-rays and 2θ is the angle between the incident beam and the scattering X-rays) was performed on at least four independent scattering trials and averaged to determine the radius of gyration (R_g_). The linearity of the Guinier region and the intensity at zero scattering angle, I(0), were used to validate that all samples were monodisperse in solution. I(0)/c, where c is concentration, was consistent for all measurements. GNOM [21] was used to determine the pair-distribution function, *P*(r), for the ERK2:STEP resting-state complex using data collected on the 1.7 mg/ml sample. Twenty-four envelopes were generated using GASBOR [22] and were aligned and averaged using the DAMAVER program suite [23].

## Results

### KIM-PTPs bind ERK2 more tightly than p38α

**Figure 1:**
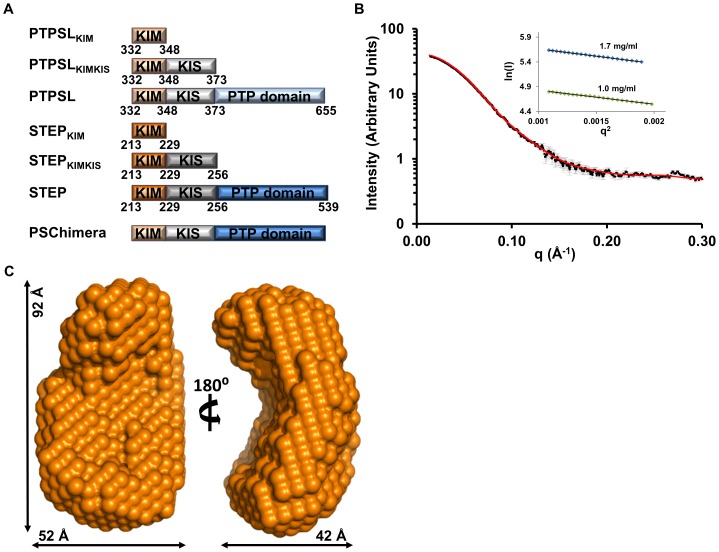
The ERK2:STEP resting-state complex. (**A**) Constructs used in this study; (**B**) SAXS data (I(q) vs q) of the ERK2:STEP resting-state complex (black squares); error bars (grey lines). Error bars show the experimental error based on circular averaging of the 2D solution scattering data; theoretical scattering curve from calculated *ab initio* molecular envelope (red); *inset*, Guinier plots for samples at 1.0 mg/ml and 1.7 mg/ml; (**C**) The ERK2:STEP *ab initio* molecular envelope in two views rotated by 90° with the dimensions of the envelope.

We used ITC measurements to determine the binding affinity of STEP and PTPSL for unphosphorylated ERK2 (**Table 1** **; Figure S1**). ITC showed that STEP binds ERK2 ∼12-times more tightly than p38α. Indeed, the ERK2:STEP complex is the tightest binding complex of all MAPK:KIM-PTP complexes tested. This complex has a ∼3-times lower K_d_ (tighter binding) than the next tightest complex, ERK2:PTPSL, and a nearly 70-fold greater affinity than the weakest complex, p38α:HePTP [15]. Like STEP, PTPSL binds ERK2 more tightly (∼3.5-fold) than p38α. We also tested peptides that include the kinase specificity sequence (KIS) regions, as we [14], [15] and others [12] have shown that KIS residues can interact directly with ERK2 and p38α. Each STEP (STEP_KIM_, STEP_KIMKIS_, STEP; **Figure 1A**) and PTPSL (PTPSL_KIM_, PTPSL_KIMKIS_, PTPSL; **Figure 1A**) domain has a higher binding affinity for ERK2 than for p38α [14]. This increased affinity for ERK2 was also observed for each domain of HePTP [15], [17]. Interestingly, the tightest p38α:KIM-PTP complex, p38α:PTPSL (K_d_∼380 nM), is equivalent to the weakest ERK2:KIM-PTP complex, ERK2:HePTP (K_d_∼370 nM) [15], [17]. Therefore, when compared to p38α, ERK2 has a higher affinity for each KIM-PTP and KIM-PTP domain (KIM, KIMKIS, KIM-PTP), showing that the increased interaction strength is clearly driven by the MAPK rather than the regulatory protein.

**Table 1:** Thermodynamic and dissociation constants for ERK2:KIM-PTP and DUSP16 MKB domains derived from ITC experiments at 25 °C.

Complex	K_d_ (nM)	ΔH (kcal·mol^−1^)	TΔS (kcal·mol^−1^)
ERK2: STEP_KIM_	546±56	−27.9±0.4	−19.4±0.3
ERK2: STEP_KIMKIS_	123±28	−29.2±0.8	−19.7±0.9
ERK2: STEP	48±20	−29.5±2.5	−19.7±2.0
ERK2: PTPSL_KIM_	463±24	−23.5±1.1	−14.9±1.1
ERK2: PTPSL_KIMKIS_	825±190	−21.6±0.8	−13.3±0.9
ERK2: PTPSL	108±36	−22.3±2.2	−12.8±2.1
ERK2: PS_chimera_	47±6	−18.5±0.8	−8.5±0.7
ERK2: DUSP16 MKBD	1303±215	−4.8±0.2	−3.2±0.3

All experiments were performed in triplicate and values reported are the experimental average and standard deviation.

We also generated a chimeric protein in which we fused the PTPSL_KIMKIS_ to STEP_CAT_ (PS_chimera_; **Figure 1A**) [14]. This was done in order to test the contribution of the STEP_CAT_ to the MAPK:protein interaction, as we previously showed that the STEP_CAT_ plays a role in binding p38α [14]. The PS_chimera_ binds marginally more tightly to p38α than any of the WT-KIM-PTPs, but binds to ERK2 with about equal binding strength as STEP (**Table 1**) [14].

### The ERK2:STEP resting-state complex is compact

**Figure 2:**
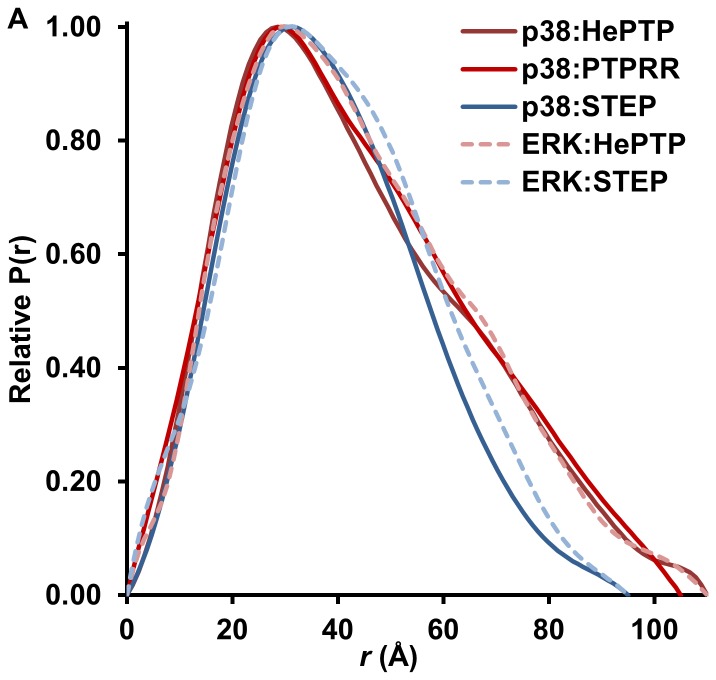
Comparison of the P(r) functions of the MAPK:KIM-PTP resting state complexes. p38α:HePTP, maroon; p38α:PTPSL, red; p38α:STEP, dark blue; ERK2:HePTP, pink; ERK2:STEP, light blue (all p38α:KIM-PTP and ERK2:HePTP data were previously published [14], [15], [17], but are shown here to provide a better comparison of the *P*(*r*) functions).

ITC measurements showed that KIM-PTPs interact more strongly with ERK2 than with p38α. In order to identify if these increased interaction strengths are correlated with structural changes we used SAXS to determine the overall shape of these complexes in solution. We recently showed that the ERK2:HePTP resting-state complex is highly extended in solution with a radius of gyration (R_g_) of 33.3 Å and a maximum dimension (D_max_) of 110 Å [16]. Here, we used SAXS to determine the size and shape of the ERK2:STEP resting-state complex (**Figure 1B**). The ERK2:STEP complex was purified by SEC and SAXS data were recorded at Beamline X9A at the National Synchrotron Light Source (Brookhaven National Laboratories). Guinier analysis of six independent SAXS samples was used to calculate an R_g_ of 30.6±0.4 Å, showing that the R_g_ of the ERK2:STEP resting-state complex is ∼3.0 Å smaller than that of the ERK2:HePTP resting-state complex (**Table 2** **;** **Figure 1C**; HePTP 324 residues; STEP 326 residues) [16]. To analyze this difference in more detail, we determined the *ab initio* molecular envelope of the ERK2:STEP resting-state complex (**Figure 1C**). A D_max_ of 95 Å was determined by analysis of the *P*(r) function, which is ∼15 Å shorter than that of the ERK2:HePTP complex (**Figure 2**) [16]. Thus, like the p38α:STEP resting-state complex [14], the ERK2:STEP resting-state complex is compact (**Figure 2**), and suggests that the STEP_CAT_ interacts directly with ERK2. Consistent with this hypothesis, STEP binds ERK2 11-times more tightly than the STEP_KIM_ peptide (**Table 1**). Based on our ITC data, both the STEP_KIS_ and the STEP_CAT_ contribute to the binding between ERK2 and STEP, as the STEP_KIMKIS_ binds to ERK2 ∼4.5-times more tightly than the STEP_KIM_ and ∼2.5-times less tightly than full-length STEP (**Table 1**).

**Table 2:** SAXS analysis of the ERK2:STEP complex.

	ERK2:STEP
**Guinier approximation**	
R_g_ (Å)	30.6±0.5
**P(r) function calculation**	
Q-range (Å^−1^)	0.013–0.303
R_g_ (Å)	31.1
D_max_ (Å)	95
**Structure modeling**	
χ^2^	2.3±0.2
NSD	1.31±0.05

The calculated R_g_ and D_max_ of the ERK2:STEP resting-state complex are highly similar to those determined for the p38α:STEP resting-state complex (**Figure 2**). Using NMR spectroscopy, we previously determined that STEP_CAT_ interacts directly with p38α; specifically, helix α-2′ of STEP binds between the p38α activation loop and the MAPK-insert [14]. Our SAXS and ITC data suggest that STEP_CAT_ also interacts with ERK2. Although there are a few structural differences between ERK2 and p38α in this region (e.g., the activation loop is six residues longer in ERK2), nearly all residues that that we showed participate in the p38α:STEP_CAT_ interaction are conserved between ERK2 and p38α [14]. Taken together, it is very likely STEP_CAT_ interacts with ERK2 via a mechanism very similar to that observed for p38α.

### The interaction of PTPSL with ERK2 and p38α is not conserved

While HePTP interacts in an extended fashion with ERK2 and p38α, our data shows that STEP associates with these MAPKs to form more compact, globular complexes. Recently, Balasu *et al*. used cross-linking, mass-spectrometry (MS) and molecular modeling to generate a structural model for the ERK2:PTPSL resting-state complex, in which they predict that the complex is compact, with ERK2 and PTPSL associating in a side-by-side manner [24]. In this model, PTPSL_CAT_ binds ERK2 in an orientation in which the catalytic site is positioned near the ERK2 phosphorylation loop. This is different from the p38α:PTPSL complex, which we showed is extended in solution [14]. Despite exhaustive attempts to collect SAXS data on the ERK2:PTPSL complex, the samples persistently exhibited the typical hallmarks of aggregation and thus, could not be used for further analysis. However, using ITC, we show that full-length PTPSL binds ERK2 ∼4-times more tightly than the PTPSL_KIM_ peptide (**Table 1**). This additional binding strength is not a result from the interaction of PTPSL_KIS_ residues with ERK2, as the PTPSL_KIMKIS_ binds less tightly than the PTPSL_KIM_. Therefore, it is the interactions between PTPSL_CAT_ and ERK2 that lead to the increased binding strength observed for the ERK2:PTPSL complex. Thus, our ITC data support the model Balasu *et al*. proposed, in which PTPSL_CAT_ directly interacts with ERK2 [24].

### Unlike the KIM-PTPs, DUSP16 binds p38α and ERK2 with similar affinities

The KIM domains of KIM-PTPs are intrinsically disordered in solution [14], [15]. Thus, they only adopt a single conformation when they bind their respective MAPKs. This is different from the MAPK-binding domains (MKBD) of MAPK phosphatases (MKP; dual specificity phosphatases; DUSPs) [25], [26]. In MKPs, the KIM sequence is part of a stable, folded domain and thus adopts a single, ordered conformation in both the unbound and MAPK-bound states [27], [28]. Using a combination of biophysical studies, we recently showed how the DUSP16 MAPK-binding domain interacts with p38α [18]. Our NMR data showed that the MAPK-binding domain of DUSP16 interacts at multiple positions with p38α. While some of them overlap with those used for KIM-PTPs (e.g. interaction with Φ_B_ and Ψ_chg_) others are distinct (helix α4) and based on our results important for the interaction of MAPK-binding domain of DUSPs with MAPKs. The ITC measurements reported a K_d_ of ∼900 nM. In order to determine if DUSP16, like the KIM-PTPs, discriminates between p38α and ERK2, we carried out similar ITC measurements between the DUSP16 MKBD and ERK2. ITC reported a K_d_ of ∼1300 nM (**Table 1** **; Figure S1**), which is only ∼1.4-fold weaker than the interaction with p38α. Thus, while the KIM-PTPs clearly exhibit an increased affinity for ERK2 over p38α, DUSP16 binds these MAPKs with similar affinities.

## Discussion

**Figure 3:**
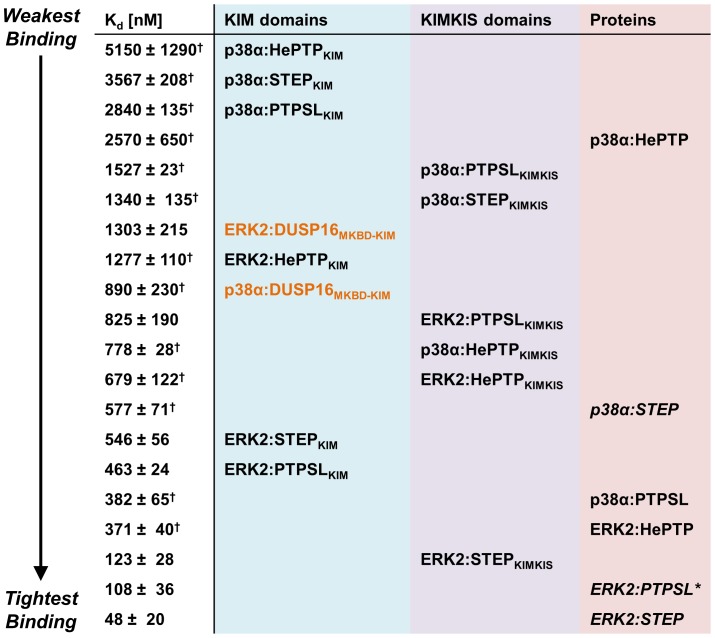
Regulatory protein-MAPK interaction strengths. The binding strengths of each KIM-PTP (KIM peptides and KIM-containing MKBDs (in orange font), light blue; KIMKIS peptides, light purple; proteins, light pink) to p38α and ERK2 represented from weakest to tightest (largest K_d_ to smallest K_d_) binding affinity; ‘^†^’ indicates values previously published [14], [15], [17], [18]. KIM-PTP:MAPK complexes determined to be extended using SAXS written in normal text; KIM-PTP:MAPK complexes determined to be compact written in italics; *, compact nature of the ERK2-PTPSL complex determined by Balasu *et al*. [24] and corroborated by ITC measurements (Table 1).

Over the last years it has become apparent that the KIM-motifs from most regulatory proteins readily discriminate between JNK and p38/ERK, however they discriminate much more poorly between p38 and ERK. For example, in a recent study, JNK-specific KIM peptides were only specific to JNK, with no detectable binding of these JNK-specific peptides by p38/ERK. In contrast, KIM peptides specific for either p38 or ERK2 exhibited discrimination factors of less than 4-fold between p38 and ERK2 [7]. Here, we show that while a subset of p38/ERK2 regulators, the KIM-PTPs, interact productively with both p38α and ERK2, their affinities for ERK2 are higher (**Figure 3**). Specifically, the KIM peptides of the KIM-PTPs exhibit differences in affinity for ERK2 versus p38α between 4.0 (HePTP) and 6.5 (STEP). Furthermore, for the interaction between MAPKs and the KIM-PTP proteins (i.e., not just the KIM-peptides), the fold differences in affinity become much larger, to as high as 12 for STEP. This shows that residues outside the KIM contribute to the overall interaction strength of MAPKs for their regulatory proteins (**Figure 3**). It also demonstrates that the increased interaction strength is clearly driven by the MAPK rather than the regulatory protein. The amino acid sequence of p38α is ∼45% identical to ERK2 and multiple residues that line the KIM hydrophobic pocket, especially around Φ_B_ (p38_ΦB_ERK2: G110E/A111T/N115K) and the CD site (p38_CD_ERK2: D124N/Q128C/E160T/D161T) are not conserved between the two MAPKs. Thus, these sequence differences are the likely reason for the reduced affinity of the regulatory proteins for p38α versus ERK2. In contrast, and more consistent with the previous study, the interaction of the KIM in DUSP16 exhibits almost no difference in affinity for p38α and ERK2, with a difference in K_d_ of only ∼1.4-fold. Together, our data shows that while KIM peptides typically poorly discriminate between ERK2 and p38α (as compared to JNK), significant discrimination for these two MAPKs can be seen for at least a subset of regulatory proteins, the KIM-PTPs. Collectively, these studies are providing a more coherent understanding of how differences in the family of KIM-PTPs with ERK2 or p38α contribute to MAPK selectively and, in turn, MAPK signal fidelity.

## Supporting Information

Figure S1 **Isothermal titration calorimetry data.** Raw isothermal titration calorimetry data (upper panels) and derived binding isotherm plotted versus the molar ratio of titrant fit using a one-site model (lower panels) for ERK2 with (**A**) PTPSL_KIM_, (**B**) PTPSL_KIMKIS_, (**C**) PTPSL, (**D**) DUSP16 MKBD, (**E**) STEP_KIM_, (**F**) STEP_KIMKIS_, (**G**) STEP and (**H**) PS_Chimera_.
(TIF)
