# Supplementary figures and images for: Interaction of Kinase-Interaction-Motif Protein Tyrosine Phosphatases with the Mitogen-Activated Protein Kinase ERK2
Source: PLoS One. 2014 Mar 17;9(3):e91934. doi: 10.1371/journal.pone.0091934 (PMC3956856; doi:10.1371/journal.pone.0091934)

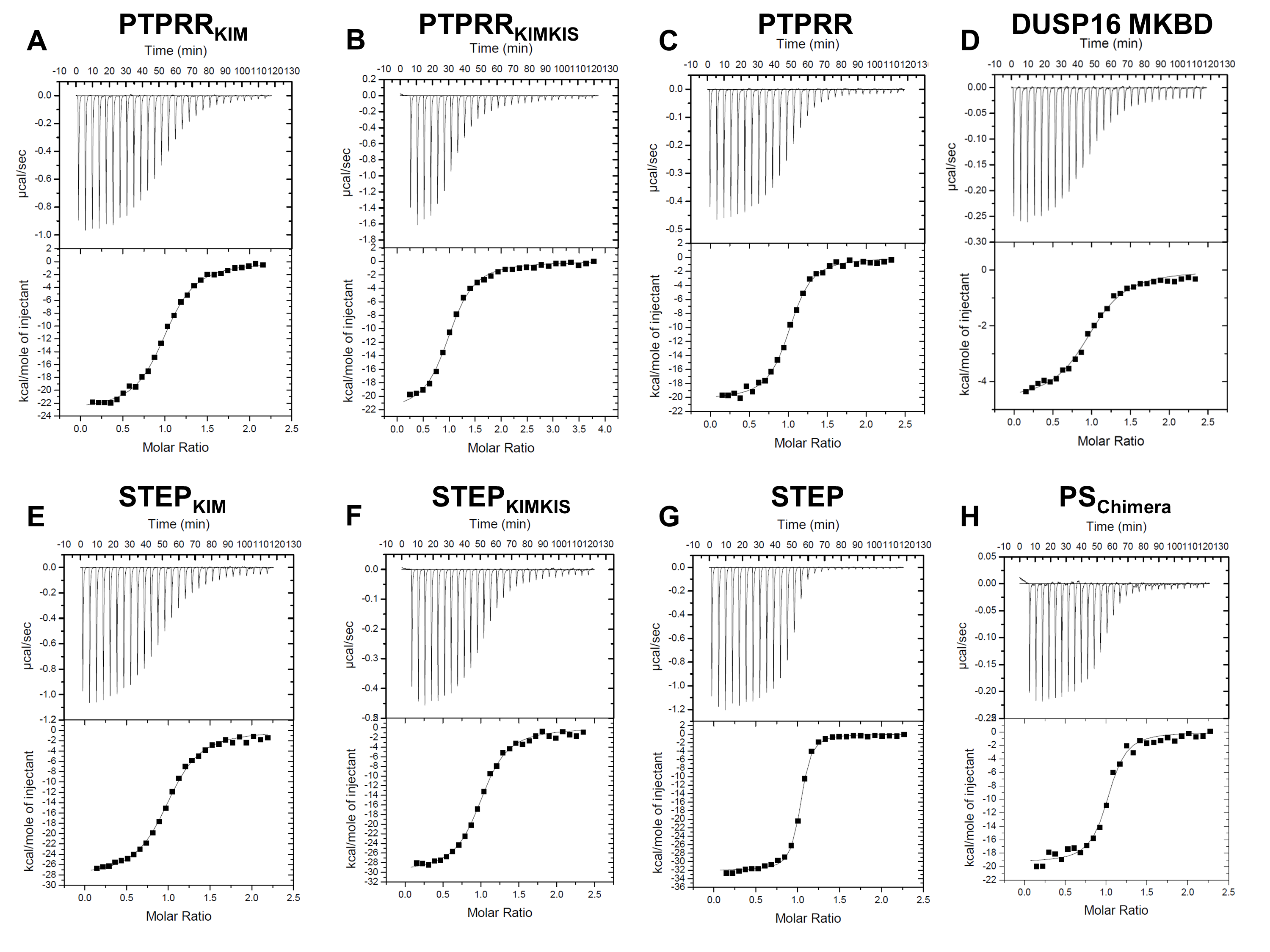

Supplement: Figure S1 — Isothermal titration calorimetry data. Raw isothermal titration calorimetry data (upper panels) and derived binding isotherm plotted versus the molar ratio of titrant fit using a one-site model (lower panels) for ERK2 with (A) PTPSLKIM, (B) PTPSLKIMKIS, (C) PTPSL, (D) DUSP16 MKBD, (E) STEPKIM, (F) STEPKIMKIS, (G) STEP and (H) PSChimera. (TIF) [file pone.0091934.s001.tif]
